# Evaluating lake water quality with a GIS-based MCDA integrated approach: a case in Konya/Karapınar

**DOI:** 10.1007/s11356-024-32184-6

**Published:** 2024-02-15

**Authors:** Ali Utku Akar, Suleyman Sisman, Harika Ulku, Esra Yel, Sukran Yalpir

**Affiliations:** 1 Department of Geomatics Engineering, Konya Technical University, Konya, Turkey; 2 Department of Geomatics Engineering, Gebze Technical University, Kocaeli, Turkey; 3 Department of Environmental Engineering, Konya Technical University, Konya, Turkey

**Keywords:** Acigol and Meke Lakes (Karapınar), Analytic hierarchy process, Geographic Information System, Lake water quality, Parameter prediction, Spatial analysis

## Abstract

Considering water quality is an essential requirement in terms of environmental planning and management. To protect and manage water resources effectively, it is necessary to develop an analytical decision-support system. In this study, a systematic approach was suggested to evaluate the lake water quality. The methodology includes the prediction of the values in different locations of the lakes from experimental data through inverse distance weighting (IDW) method, creation of maps by using Geographic Information System (GIS) integrated with analytic hierarchy process (AHP) from multi-criteria decision analysis (MCDA), reclassification into five class, combining the time-related spatial data into a single map to predict the whole lake water quality from the data of sampling points, and finally overlapping the final maps with topography/geology and land use. The proposed approach was verified and presented as case study for Meke and Acigol Lakes in Konya/Turkey which were affected by human and natural factors although they have ecological, hydromorphological, and socio-economic importance. In the proposed approach, categorizing water quality parameters as “hardness and minerals,” “substrates and nutrients,” “solids content,” “metals,” and “oil-grease” groups was helpful for AHP with the determined group weights of 0.484, 0.310, 0.029, and 0.046, respectively. Assigning weights within each group and then assigning weights between groups resulted in creating accurate final map. The proposed approach is flexible and applicable to any lake water quality data; even with a limited number of data, the whole lake water quality maps could be created for assessment.

## Introduction

The surface water quality is degraded due to widespread pollution caused by various anthropogenic activities, including rapid population growth, mining activities, uncontrolled tourism activities, unplanned human settlement, deforestation, intensive farming, animal husbandry, and encroachment into wetlands. These activities directly or indirectly cause water pollution in the basin and ultimately in the coastal region of the lake. Therefore, programs should be established in order to prevent pollution, protect ecosystem integrity, and improve water quality on a basin basis. Environmental measures including pollution control, clean production, and the best techniques should be taken for point and diffuse sources of pollution in terrestrial-wetland areas. Before taking a concrete environmental step, it is necessary to evaluate the pollution sources and hydromorphological factors affecting the water body.

Lake water is one of the main concerns of a range of hydrogeochemical processes such as evaporation, mixing, precipitation, and dissolution of minerals that occur between water, sediment, and atmosphere (Njenga 2004; Davraz et al. 2019). Water quantity and quality of both lakes and feeding streams may vary depending on ecosystem components, anthropogenic effects, geological structure, topography, land use, as well as climate change, erosio,n and decomposition of crustal materials (Sahoo et al. 2013; Mallick 2017; Davraz et al. 2019). Therefore, lake water quality has been studied extensively (Jeelani and Shah 2006; Varol et al. 2012; Tao et al. 2013; Şener et al. 2014; Mohamed et al. 2015; Li et al. 2017; Wu et al. 2017).

Information technologies have a great contribution to the protection, development, and evaluation of water resources. The methods used in water quality assessment and frequently encountered in the literature are complex pollution indexes (CPI) (Wei et al. 2009), water quality index (WQI) (Ram et al. 2021), principal component analysis (PCA) (Tokatlı and Varol 2021), artificial neural network (ANN) (Jiang et al. 2006), and fuzzy comprehensive evaluation (FCE) (Liu et al. 2010). However, these methods do not consider the spatial continuity of the sampling data, which can limit their effectiveness in certain applications. On the other hand, it is difficult to interpret the situation of the lake without referring to the land with its surrounding use functions, including direct or indirect human influence. In addition to examining multiple factors for water quality assessment in regional studies, the spatial distribution of pollution levels should also be taken into account. In this context, Geographic Information System (GIS) becomes important tool to manage water resources (Kalıpcı et al. 2017; Zeydan et al. 2019). GIS is a decision support mechanism that performs spatial analysis/modeling and offers the opportunity to evaluate spatial data. It is also indispensable for optimizing the management of natural/artificial resources and land use planning. The use of GIS for sustainable land management adds privilege to the work.

In many studies, water quality has been evaluated with the help of GIS analyses by using the data obtained from analyses of samples from water sources (Zahran et al. 2015; Jabbar et al. 2019; Huang and Tian 2019; Vasistha and Ganguly 2022). In addition to the analyses, there are also studies that examine water quality by considering the environmental factors around the water source (Al-Adamat 2017; Avram et al. 2021; Long et al. 2021). Geostatistical analysis and GIS were integrated to visualize spatial models of water pollution/quality and estimate values for unknown locations (Yan et al. 2015; Mir et al. 2017). GIS and spatial modeling have long been preferred in watershed-scale environmental pollution studies and risk assessment for land use (Chang 2008; Jasmin and Mallikarjuna 2014; Yuan et al. 2020).

Water-quality evaluation studies should be carried out not by examining only parameters separately, but by considering all the parameters together. The simultaneous evaluation of all parameters often gives more meaningful results and enables different analyses to be performed in the decision-making process. On the other hand, there are various difficulties in working with multiple parameters. Deciding the importance degree and weight effect of the parameters is one of these difficulties. In order to make an effective evaluation of water quality, a methodology that combines physical, chemical, and biological parameters should be applied. Considering that the quality classes consisting of different groups also contain sub-parameters, it will be possible to evaluate the main and subclasses together in an easier and more understandable way by using the analytic hierarchy process (AHP), one of the multi-criteria decision analysis (MCDA) methods. The AHP has been used in many applications to evaluate the importance of multiple criteria in the field of environmental and earth sciences (Barış et al. 2007; Jabbar et al. 2019).

The AHP assists decision-makers in evaluating priorities among different criteria and making decisions among alternatives based on these priorities. This method is particularly used in complex and multifactor decision-making problems, providing an analytical and structured approach (Saaty 1977). The AHP can be combined with GIS in decision making. In addition to the spatial and temporal evaluation of data, GIS also provides benefits for monitoring water quality changes or identifying potential sites (Pang et al. 2008; Rawat and Singh 2018; Chabuk et al. 2020). In this regard, GIS-AHP is a procedure in which valuable information can be obtained through the use of spatial and non-spatial data that can assist in critical decision making based on the judgment of decision makers (Gbanie et al. 2013; Kamdar et al. 2019). It can significantly contribute to the research of water quality, conservation of natural resources, and preparation of the management plan (Saha and Paul 2021).

The aim of this study is to provide an approach to generate the water quality maps from small amount of data to estimate the water status of a lake and to relate the approximate status with environmental conditions of lake. The proposed approach was applied to collected lake water data from the Acigol and Meke Lakes. The purpose of studying geographically close two lakes is both to investigate the reliability of the approach on more than one lake data and to relate the same geographical and environmental conditions to the predicted water qualities coparatively. In the proposed approach the spatial distribution of laboratory analysis findings obtained from a limited number of sampling points has been identified. Additionally, the water-quality parameter maps were grouped and indexed. For indexing, weighting was made by using AHP and thus water quality maps of both lakes were generated. This study will offer a flexible decision support system for water-quality evaluation roughly for all water bodies in a similar way through two specific lake examples. By this proposed approach, deriving the whole lake water quality map from less number of analytical data will have indirect economical importance as this will reduce the sample collection and analysis costs.

## Materials and methods

### The study area

Konya Closed Basin (KCB) is in the Central Anatolia Region and is a flat plain at an altitude of 900–1050 m, covering 7% (~ 50,000 km^2^) of Turkey’s surface area. The high mountains formed of volcanic rocks and limestone surrounding the KCB, which is the largest closed basin in Turkey, also prevent drainage to the sea, and its soil is generally salty as a result of insufficient drainage (Bozyiğit and Güngör 2011). The basin has a continental climate with hot and dry summers and cold and rainy winters, with an annual average precipitation of 287 mm. But because of high soil permeability the water depth has never become high.

**Fig. 1:**
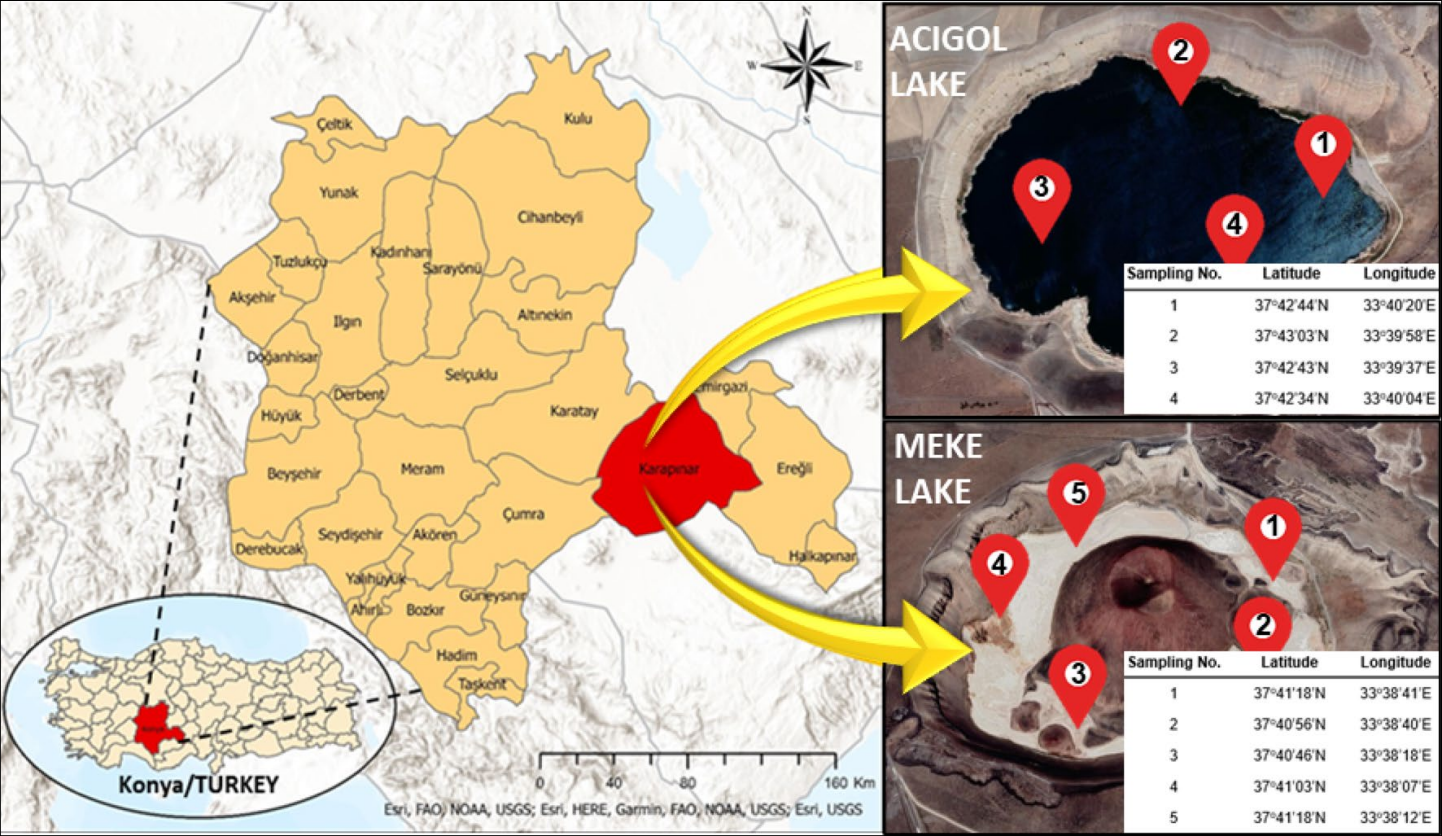
Location of the Acigol and Meke Lakes and sampling points

The study area is Acigol and Meke Lakes located in the KCB (Fig. 1). Young volcanic formations are common in the southern and eastern parts of Karapınar. Six out of 10 known “Maars” (volcanic ash pit accumulated around the crater as a result of volcanic activities) in Turkey are in this area. The most well known among these are the Meke and Acigol Maars. Meke is a crater lake with islets in the middle, formed by the filling of an extinct volcano crater with water as a result of two-stage volcanic activity. Acigol, another crater lake-like maar, consists of a shallow volcanic eruption pit. The environment of the lakes is the breeding area of bird species and there is no life in the water except for microbiological activity (Arık et al. 2012). The quality of the lake water is important for the protection of the geological structures that took millions of years to form, for the geopark tourism that has become widespread in the world and for the land use activities around it. These lakes, which are privileged natural resources with these qualities in Turkey, were selected as the study area as they have both socio-economical and ecological importance.

The natural features of the region make it socio-economically important; however, it has a drought problem in summer due to the spread of irrigated agriculture and the decrease in the groundwater level. Depending on global climate conditions, Acigol and Meke Lakes dry up in some years and partially revive with rainfall in some years. The KCB and its surroundings, where the lakes are located, are frequently brought to the agenda with the formation of sinkholes. In this region, which serves photography and nature tourism, work has been initiated to include lakes and potholes in the UNESCO Global Geopark Network (URL1 n.d). Meke Maar and its surroundings are the first-degree natural protected area. In addition, 260 hectares of it has the status of a natural monument, and 202 hectares of it was declared as Ramsar Area in 2005 (Tunçez and Candan 2008).

**Fig. 2:**
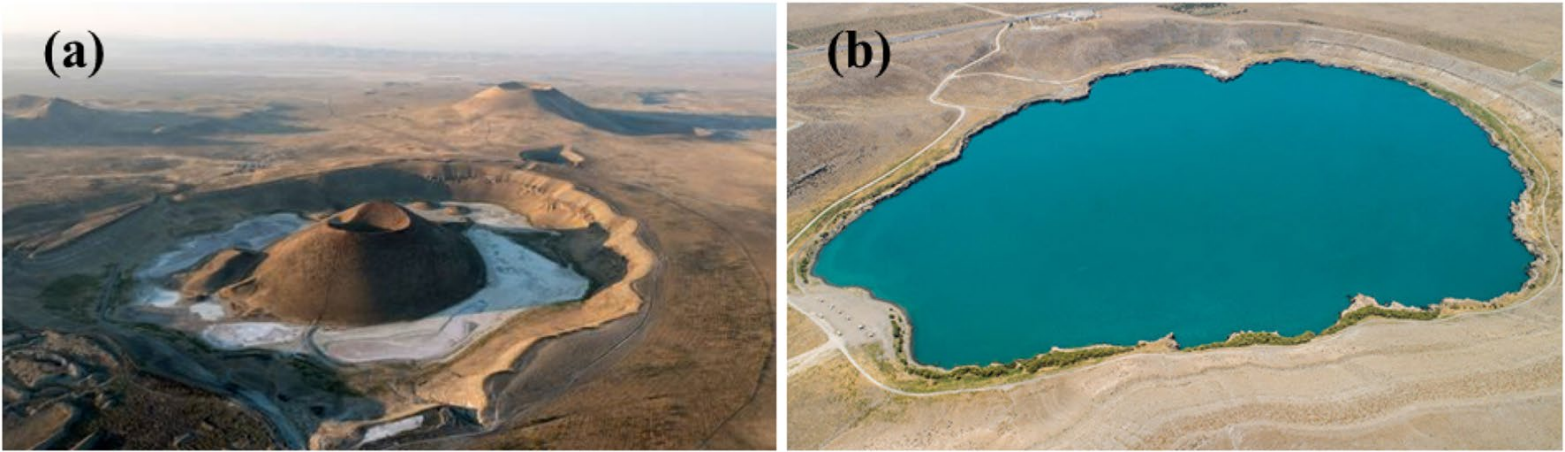
**a** Meke Maar and **b** Acigol Lake (URL2 n.d)

Meke Lake, also known as the Evil Eye bead, is located approximately 7–8 km southeast of Karapınar. The altitude of Meke Lake is 981 m, its length is 800 m, and width is 500 m. The water depth of the lake depends on the level of the underground water table. While its surface area was 0.5 km^2^ and depth was 12 m in the year 2000. The maximum depth decreased to 1.5 m in the subsequent years when it was not completely dry (Fig. 2a) (Maleki 2013). Meke Lake is one of the rare lakes in the world in terms of its formation. It has a similar formation story to the Ngorongoro Caldera in Africa, which is the largest caldera in the world. It formed as a result of two separate volcanic eruptions about 4 million years ago from the Pleistocene. The crater, formed by the first eruption with a diameter of about 4 km, filled with water over time and became a lake. A pyroclastic volcanic cone in the lake and the volcanic Meke Hill with 50 m height in the middle of the lake were formed after a second eruption about 8000 years ago. The middle of the hill is sunken in. In the later stages of the formations, seven small hills called “Parasitic Cone” were formed by various eruptions. Some of the hills are adjacent to the main cone and some of them are in the form of islands.

Inandik (1965) reported that there were springs at the bottom of Acigol and Meke Lakes and he attributed the salinization of the lakes to the spring waters rich in K, Mg, Na, Ca sulfate, and chloride. For centuries, the salts in the incoming waters accumulated in the lake pits due to the evaporation of water and the salt content of the lakes gradually increased. The salt rate of Meke Lake can reach up to 32% in very shallow and dry seasons (Aycan 2012). Salt (NaCl) was produced from the lake for a period when the water level dropped and salt layers were formed in the parts dried by evaporation (Fig. 2a).

Acigol Lake, which is a wide and slightly swollen crater, is a lake that was formed as a result of a large volcanic eruption caused by the volcanism that started in the Miocene and continued in the Quartener. The area around the lake is covered with steep slopes and volcanic ash. The lake is fed from underground. The water tastes bitter due to its salty and carbonated content (Akkoz et al. 2009). The long axis of Acigol Lake in the northwest-southeast direction is 1750 m and the short axis is 1250 m. Its altitude is 985 m and surface area is 1.2 km^2^ (Fig. 2b) (Maleki 2013). At the beginning of the 1990s, its depth was reported to be 100 m (Biricik 2014), and in the following years, its depth was 3 m (Aycan 2012). Pyroclastic material is widespread around the crater and the water of the lake is salty as in Meke Lake; it cannot discharge its water to the outside (Akdemir 2004).

### Methodology

Five sampling points on Meke Lake and four sampling points on Acigol Lake were chosen (Fig. 1). As the water depth and lake surface area were low, the selected sampling points were representing the whole lake when considering the grids. Moreover, as the main aim of the study was to achieve an estimation of lake water status by producing maps from limited number of data, the number of sampling points was kept as low as possible. Sampling depth was a nominal depth of approximately 20 cm and only that level was considered; water depth was not studied as independent variable. Sampling was performed in 2015 monthly for 1 year, but there was icy season and complete dry season with no water in the lakes, and this resulted in 10 sampling periods from each sampling point of each lake in a year. Thus, a total of 90 samples, 50 from Meke Lake and 40 from Acigol Lake, were obtained and water quality parameters in these samples were analyzed according to standard methods (APHA 2008). Within each sample, 22 water quality parameters were considered. Eighteen parameters were categorized into five groups: “hardness and minerals,” “substrates and nutrients,” “solids content,” “metals,” “oil-grease content,” and those considered “excluded parameters.”

1. Hardness and minerals: acidity, alkalinity, ca hardness, total hardness, chloride (Cl^−^) and sulfate (SO_4_^2−^).
2. Substrates and nutrients: chemical oxygen demand (COD), biochemical oxygen demand (BOD_5_), total Kjeldahl nitrogen (TKN), total phosphorus (TP), and chlorophyll*-a* (Chl*-a*).
3. Solid content: turbidity, suspended solids (Ss), and total solids (Ts).
4. Metals: chromium (Cr), copper (Cu), and nickel (Ni).
5. Oil-grease content: oil and grease (O–G).
6. Excluded parameters: pH, dissolved oxygen (DO), ammonia nitrogen (NH_3_–N), and organic matter. pH and DO had almost no spatial and seasonal variations. For example, pH was in the range of 7–8 and DO value was in the range of 0–0.7 mg/L. They would not provide effective findings in decision making with a spatial change detection. Moreover, NH_3_–N values were included in the TKN parameter, and organic matter is represented by BOD_5_ and COD parameters; therefore, NH_3_–N and organic matter were analyzed as a kind of control parameter. Therefore, all these parameters were not included in the modeling study.

**Fig. 3:**
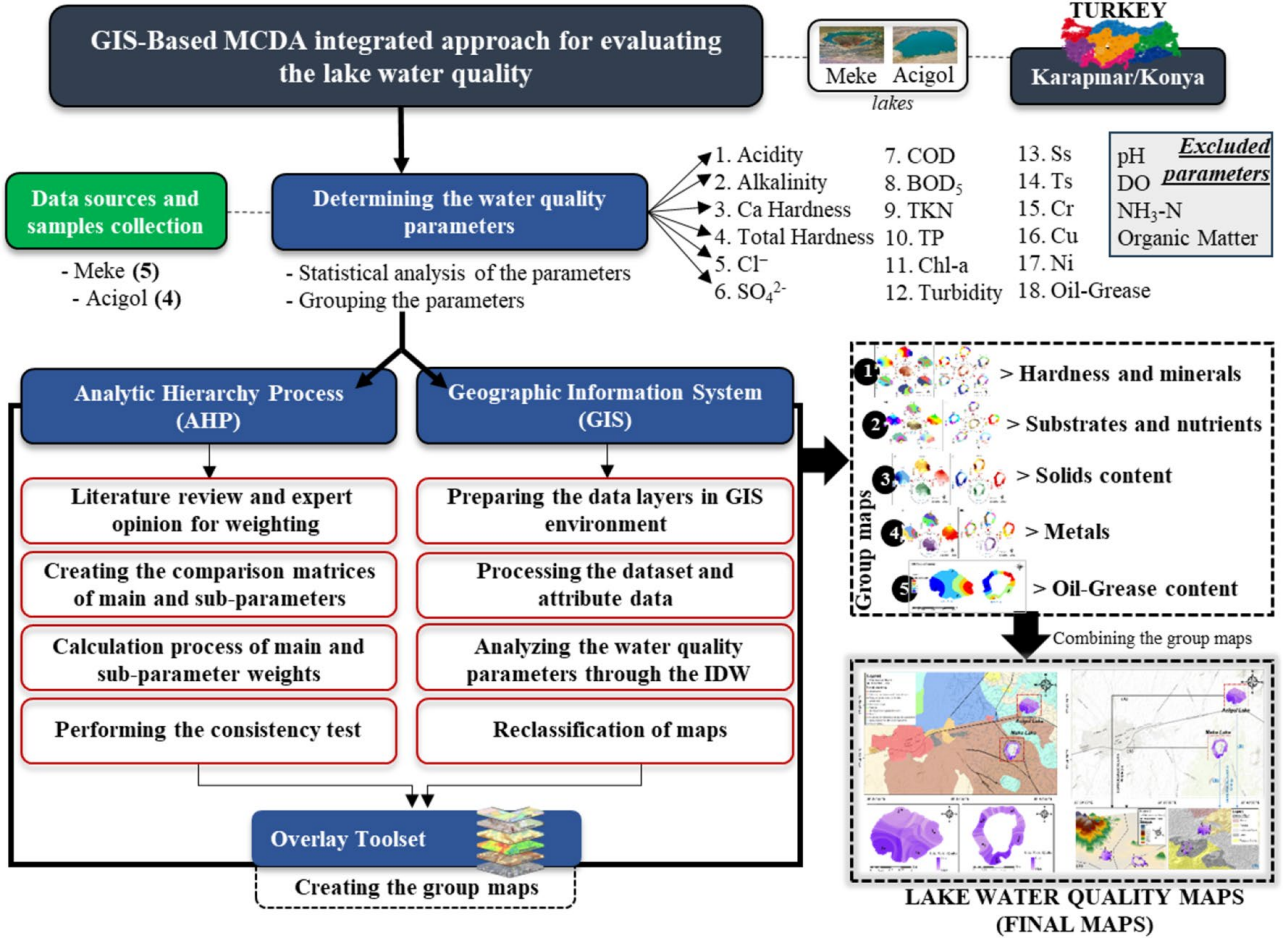
Methodological framework of the study

The designed framework in this study is presented in Fig. 3. Groups and parameters of water quality were weighted by expert opinion for AHP method. The given weights were used in GIS for lake water–quality mapping for each parameter, then for each group, and the integrated water quality maps were obtained for each lake. Finally, these maps were discussed and were related to the topographical, environmental, geological conditions, and land use.

### Applying analytic hierarchy process (AHP) for lake water quality parameters

AHP is a theory of measurement through pairwise comparisons and relies on the judgments of experts to derive priority scales. It is important to organize these decisions to set priorities and the organizational chart needs to decompose into the following steps (Saaty 1980).

- i. Identify the problem and decide the type of needed information (determining and weighting the effecting parameters for the lake water quality map in this study).
- ii. Structure the decision hierarchy from the top with the aim of the decision, then the targets from a broad perspective, through the intermediate levels to the lowest level (hardness and minerals, substrates and nutrients, solids content, metals, oil-grease content, and sub-parameters of these groups).
- iii. Construct a set of pairwise comparison matrices (Eq. 1). Each parameter in an upper level is used to compare the parameters in the level immediately below it (hardness and minerals 6 × 6, substrates and nutrients 5 × 5, solid content 3 × 3, metals 3 × 3, and oil-grease content 1 × 1).
  1 $$A=\left[{a}_{ij}\right]=\left[\begin{array}{ccccc}1& {a}_{12}& \dots & \dots & {a}_{1n}\\ 1/{a}_{12}& 1& \dots & \dots & \dots \\ \dots & \dots & 1& \dots & \dots \\ \dots & \dots & \dots & 1& \dots \\ 1/{a}_{1n}& \dots & \dots & \dots & {a}_{nn}\end{array}\right]$$
  2 $${{a}^{*}}_{ij}=\frac{{a}_{ij}}{\sum_{i=1}^{n}{a}_{ij}}$$
  3 $${w}_{i}=\frac{\sum\limits_{j=1}^{n}{a}_{ij}^{*}}{n}$$
  ${a}_{ij}$, matrix elements;* n*, number of parameters, *i, j:* 1, 2, 3…n.
- iv. Use the priorities obtained from the comparisons to weigh the priorities in the level immediately below (Eq. 2). Then for each element in the level below, add its weighted values and obtain its overall priority (Eq. 3). Scale values should be given to the parameters according to their importance. These values are made by experts in the field. In this study, scale values were given by authors/academicians who work as experts in Turkey.
- v. The matrix of pairwise comparisons *A* = [*a*_*ij*_] represents the intensities of the expert’s preference between individual pairs of alternatives (Alonso and Lamata 2006). Consistency ratio (CR) is calculated to check the consistency of these matrices. CR is found using CR = CI/RI formula, where RI is the random index in Table 1. Consistency index (CI) is found using CI = (*λ*_max_ − *n*)/(*n* − 1) formula, where *λ*_max_ is the eigenvalue and *n* is the matrix size. If CR is calculated as CR ≤ 0.10, the assessment is consistent. However, if CR is calculated as CR > 0.10, the assessment is not consistent and thus must be restructured.

**Table 1:** Random index (Saaty 1980)

*n*	1	2	3	4	5	6	7	8	9	10	11	12	13	14	15
RI	0.00	0.00	0.58	0.90	1.12	1.24	1.32	1.41	1.45	1.49	1.51	1.48	1.56	1.57	1.59

### GIS mapping process

This study included measurements of five sampling points in Meke Lake and four sampling points in Acigol Lake. The results of analyses of these samples were arranged as Excel file; data layers were created in the GIS. Inverse distance weighting (IDW) method was used for interpolation. IDW analyses were performed using Spatial Analyst toolbox of ArcGIS 10.5.1. IDW is an interpolation method that estimates point values using the mean values of sample data points near each point. The general equation of the IDW method used in the study was as follows (Eq. 4):

4 $$\widehat{Z}({x}_{0})=\sum_{i=1}^{n}Z\left({x}_{i}\right).{d}_{ij}^{-p}/\sum_{i=1}^{n}{d}_{ij}^{-p}$$

where $\widehat{Z}({x}_{0}$) is unknown value, *n* is the number of points taken to obtain the unknown value, $Z\left({x}_{i}\right)$ is the *i*th known value, ${d}_{ij}^{-p}$ is distance between the *i*th unknown value and the *j*th known value, and *p* is the power (ESRI 2022).

In the study, first, the parameter variation at different locations of the lake was analyzed by means of IDW using the measured values at the points. From the results of the analyses, time- and location-based variation maps in vector format were produced for each parameter as the average of analytical result of 10 periods (in Fig. 7 the temporal and spatial variation of the acidity parameter in Acigol Lake was given as an example, these maps were created for all parameters in the study). The maps were converted into raster maps, and they were integrated via reclassification and overlay analyses (i.e., group maps created). Reclassification is a normalization process that enables the classification of raster data according to user requests. It was applied to be used as input data in the analyses to be made in the next stage. In reclassing rasters, the existing raster creates two effects. The first is the conversion of rasters with decimal values in their cells to integers, and the second is that the created classes can be used in desired ranges. The data converted to raster format as a result of these operations can then be used in many mathematical functions and overlay operations since they consist of cells (ESRI 2022). In this context, the reclassified maps were prepared by classifying in five equal intervals and assigning a value of (1) to the class with the lowest parameter value and (5) to the class with the highest parameter value. Here, the low parameter values represent high water quality, and high parameter values vice versa. Finally, the lake water–quality maps/final maps were generated by integrating the grouped maps. Overlay analysis was used for applying a common scale of values to diverse and dissimilar inputs to create an integrated analysis. The threshold values in the related Regulation of the Ministry of Environment, Urbanization and Climate Change were taken into account in the analyses (WPCR 2004).

## Results and discussion

### Statistical analysis of lake water parameters

**Fig. 4:**
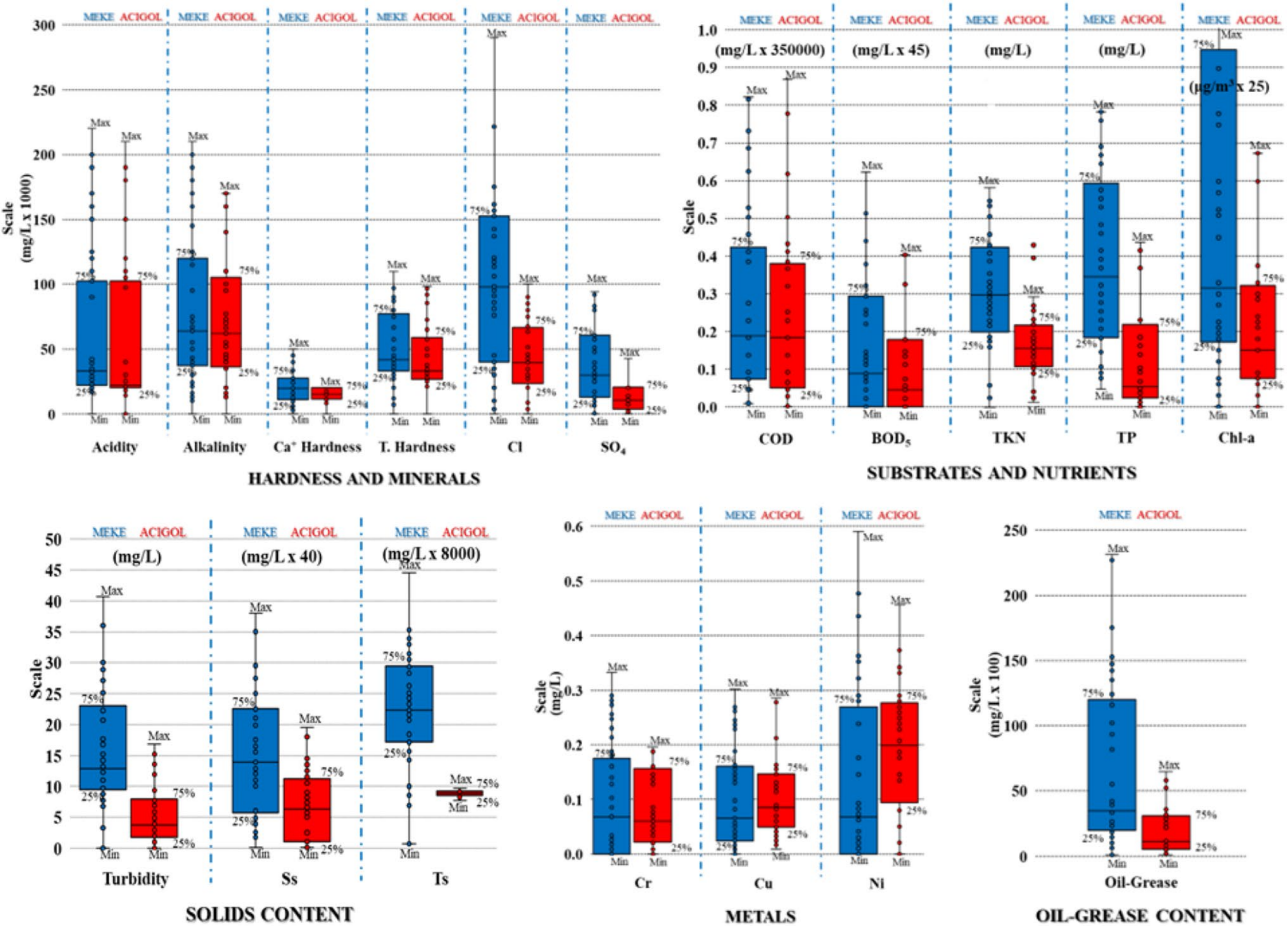
Box-Wilcox diagrams of detected water quality parameters in Acigol and Meke Lakes

Lake-water quality may contain different parametric properties according to the origin and classification of the lake. Some lakes are fed by precipitation and clean groundwater, and are clear and potable, while others are formed by volcanic eruptions and have a high salt content. Different physical, physicochemical, and biological parameters were examined in order to evaluate the water quality according to the dissolved or particulate components, or physical and chemical quality parameters in the water (Bhateria and Jain 2016). Laboratory analyses of physicochemical parameters were carried out to determine the quality of the water obtained from the sampling points for the two lakes. As stated before, four parameters were grouped as “excluded parameters.” The remaining parameters were divided into five main groups according to their characteristics to be used in water quality evaluation (Fig. 4).

**Fig. 5:**
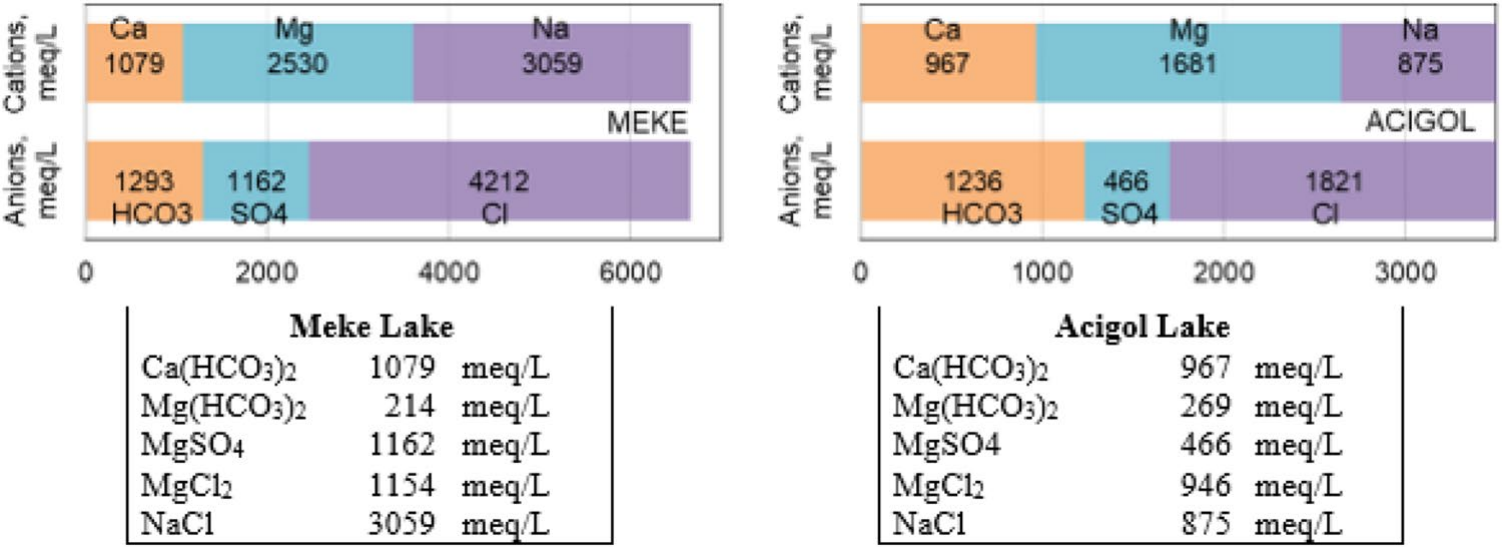
Bar diagrams for identifying hardness compounds and concentrations

Simultaneously high acidity and alkalinity in the “hardness and minerals” group was an important indicator representing the presence of both carbonate and non-carbonate hardness (Fig. 4). On the other hand, while the Ca concentrations were low, the high total hardness indicated the excess of Mg-induced hardness compounds, and the high acidity, SO_4_^2−^ and Cl^−^ ions indicated that Mg-induced non-carbonate hardness was dominant. To illustrate this situation more clearly, bar diagrams were created with the annual average data at all stations for both lake waters in Fig. 5. The concentrations of parameters in all “hardness and minerals” group in Meke Lake were higher than Acigol Lake. The concentrations of almost all compounds detected according to the bar diagrams were also higher; only Mg(HCO_3_)_2_ was slightly lower in Meke Lake (Fig. 5). Ca^+2^ ion can originate from the dissociation of salts, such as calcium chloride or calcium sulfate, and it is one of the major inorganic cations, in saltwater and freshwater. CaCO_3_, limestone, gypsum, and other calcium-containing rocks are the main sources of Ca^+2^ in water (Bhateria and Jain 2016).

In the literature, Meke and Acigol waters were defined as Na–Cl–HCO_3−_-type waters. The concentration of HCO_3−_ ions was approximately the same in both, whereas SO_4_^2−^ and Cl^−^ were higher in Meke Lake. Most of the SO_4_^2−^ compounds in water originate from the oxidation of sulphate ores, the solution of gypsum and anhydrite, the presence of shale, particularly those rich in organic compounds, and the existence of industrial wastes (Bhateria and Jain 2016). Sulfur-bearing minerals are common in travertine rocks. In the weathering process, gypsum dissolve and sulfide minerals are partially oxidized, giving rise to a soluble form of sulfate that is carried away by water. In semi*-*arid and arid regions, the soluble SO_4_^2−^ salts may accumulate (APHA 2008). The CO_2_ gas coming from the deep dissolves in the groundwater to form H_2_CO_3_, and then the H_2_CO_3_, H^+^ ion decomposes into the HCO_3_ ion (Nazik et al. 2004). This also lowers the pH value in groundwater. In addition, HCO_3_ is also added to the groundwater as a result of the dissolution of limestone. SO_4_^2−^ ion, on the other hand, is formed by the dissolution of gypsum levels in lacustrine sediments in groundwater. Similar to the findings in this study, 0.3 g Ca^+2^, 120 g Mg^+2^, 200 g SO_4_^2−^, and 188 g Cl^−^ were detected in 1 L of Meke Lake water (Karaaslan 2010).

Three parameters, turbidity, Ts, and Ss, were classified in “solids content” group (Fig. 4). Solids are found in streams in colloidal, suspended, dissolved, and volatile. Colloidal particles cause turbidity which directly affects the light penetration into the surface water bodies. Ss include silt, sand, decaying plant matter, or sewage treatment effluent. Fertilizers from fields and lawns can add a variety of ions to a stream (Bhateria and Jain 2016). Ts is directly proportional to the TDS. In areas of especially hard water or high salinity, TDS values may be as high as 500 mg/L. Meke Lake water turbidity, Ss, and Ts levels were considerably higher than Acigol Lake water. This had first related to the water depth, such that Acigol Lake is much deeper and stirred-up bottom sediment does not cause solids in water, whereas Meke Lake is so shallow that water is always turbid due to both sediments and other factors such as salts.

The main reason for selecting the parameters in “substrates and nutrients” group is both to indicate whether there is organic pollution in the water bodies and to investigate the possible microbiological life in the lakes. Therefore, this group includes substrates (BOD_5_ and COD), nutrients (TKN and TP), as well as Chl*-a*, which represent the algal population. There is no bacterial ecosystem in these lakes due to mineral content; however, some algal species can survive. In this context, nutrients were related to the algal population, while substrates may rather indicate organic pollution. The greater the BOD_5_, the more rapidly oxygen is depleted. Sources of BOD_5_ include leaves and woody debris, dead plants and animals, animal manure, industrial effluents, wastewater treatment plants, feedlots and food-processing plants, failing septic systems, and urban stormwater runoff (Bhateria and Jain 2016). There are many sources of phosphorus, both natural and anthropogenic. These include soil and rocks, wastewater treatment plants, runoff from fertilized lawns and cropland, failing septic systems, runoff from animal manure storage areas, disturbed land areas, drained wetlands, water treatment, and commercial cleaning preparations. In the nature, phosphorus usually exists as organic and inorganic. Both can either be dissolved in the water or suspended attached to particles in the water column (Spellman 2014). Algae adjust their Chl*-a*/C ratios based on P availability (Abirhire et al. 2015). The concentrations of all parameters in “substrates and nutrients” group in Meke Lake water were higher than in Acigol Lake water (Fig. 4). As compared to each other, BOD_5_ and COD levels of both lake waters were closer, whereas TKN and TP concentrations in Meke Lake water were much higher than in Acigol Lake water. The large difference in nutrient content despite the closeness of the organic contents explains the large difference in Chl*-a* concentrations between the two lake waters. In addition to nitrogen and phosphorus, an inorganic carbon source, which is provided by HCO_3_ ions, was sufficient for algae, which are photosynthetic organisms. Among the findings in this group, BOD_5_/COD ratios between 0.4 and 0.7 indicated that there is organic pollution in the water. The findings of the oil-grease parameter evaluated in a different group also support this (Fig. 4).

Cr, Cu, and Ni were detected very close to each other in Meke and Acigol waters at very low concentrations. As in other parameters, there is no significant difference between the waters of the two lakes (Fig. 4). The findings of this evaluation made with the average values of all detections in the lake waters showed that the pressure and impacts of the region on the lake waters are mostly of organic (anthropogenic) or hydrogeological origin. For this reason, spatial maps were obtained by spatial evaluation of the quality of lake waters with separate values at sampling points.

Since Meke Lake is in a closed basin, it cannot take the advantage of snow and rain water. It was observed that other lakes and wetlands around it were also drying up. Akgol Lake, located between Ereğli and Karapınar in the south of Meke Lake, has become completely used as agricultural land. The reasons for the drying up of Meke Lake include unconscious agricultural irrigation, excessive use of groundwater in the KCB, the drought in Turkey caused by climate change, and the constant water loss because of evaporation of the water on the lake surface. Crystallization and salinization occur as a result of evaporation of the small amount of water remaining due to drought in a large part of Meke Lake, which has a crater structure (Akkoz et al. 2009).

There is a direct relationship between precipitation and groundwater budget. Generally, groundwater levels increase in winter and spring months when precipitation is high during the year, and decrease in summer when precipitation is very low. Across the KCB, there is a significant lowering of the water table as a result of excessive water withdrawal from approximately 94,000 wells to grow water-demanding crops (sugar beet, maize, sunflower, vegetables, alfalfa, etc.) and increasing drought (Doğdu et al. 2007; Bulduk et al. 2008; Bayari et al. 2009; Yılmaz 2010). Göçmez et al. (2008) stated that 60% of the groundwater changes in Konya and 40% in Karapınar were related to climatic variables and the rest was related to excessive water withdrawal which caused the groundwater level in the basin to decrease and some aquifers to lose their properties. As a result, many marshes and springs dried up. Decreases in water level of both Acigol and Meke Lakes were observed on a yearly basis. According to the water level measurements made by DSI (1975), while the static water level altitude in the slope sinkhole was 986 m, it decreased to 964.53 m in 2009, which means there was a 21.5-m water level drop. In the next periods, although the water level of Meke Lake was seen in May, there was no water in Meke Lake in August. While the underground water reserve was 2418.50 hm^3^/year in 2013 data for KCB, it was observed that it decreased to 2023.00 hm^3^/year in 2019 data (URL3 n.d).

**Fig. 6:**
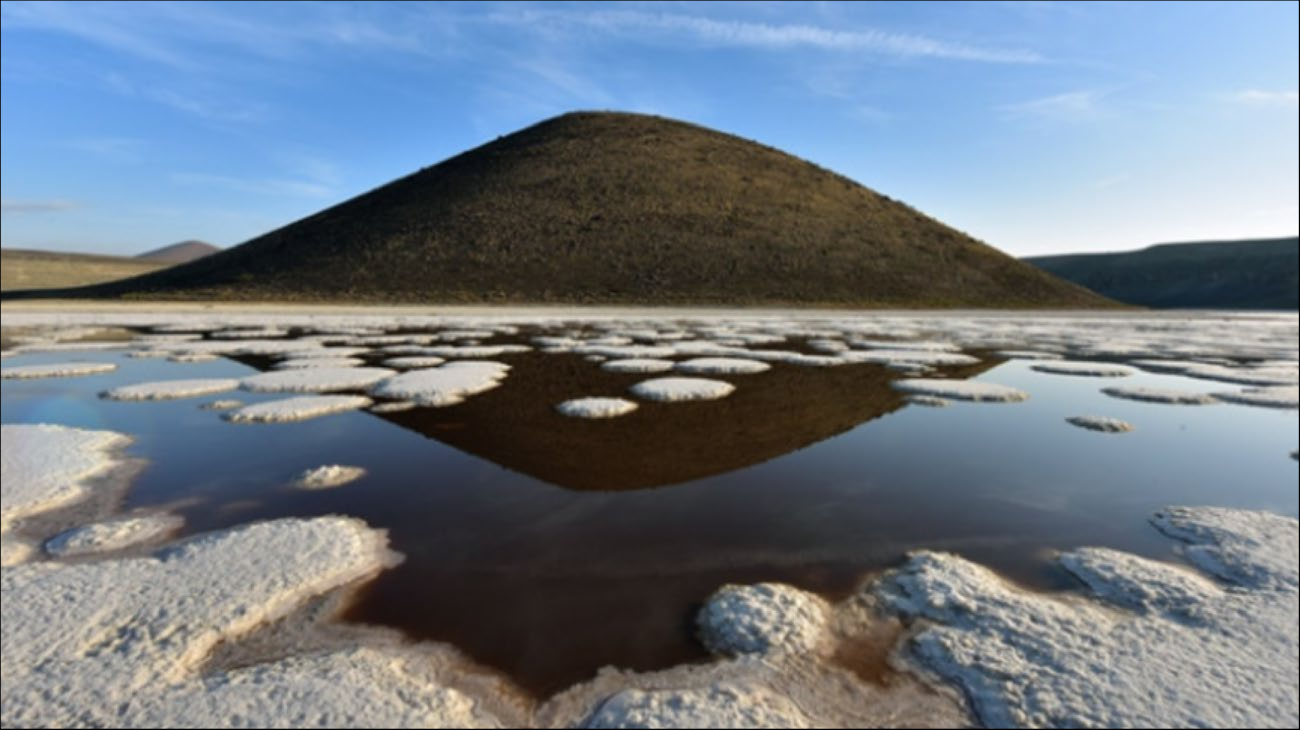
Foaming due to pollution in Meke Lake water (URL1 n.d: 2016)

In the 2000s, foaming began to be seen in the water surface of Meke Lake due to pollution (Fig. 6). The bottom of the lake, where islets were formed, started to become muddy. Since such foaming is not expected to occur considering the hydrogeological structure of the lake; this situation is likely to be caused by pollution in the lake water. It is predicted that free movement around the lake and the presence of agricultural lands and other human activities (transport, etc.) in the immediate vicinity are effective on pollution.

### Weighting of the parameters

Each water quality parameter was named as “sub-parameter,” while parameter groups were named as “main parameters.” A total of 5 main parameters, 1) hardness and minerals, 2) substrates and nutrients, 3) solid content, 4) metals, 5) oil-grease content, and 18 sub-parameters of these were discussed in the evaluation of lake water quality. In this section, an example of method application for the sub-parameters of the “hardness and minerals” group is presented (Table 2). Weights were calculated using Expert Choice software. After the parameter weights were determined, a consistency test was applied to validate the results. As a result of the consistency test, the CR value was found to be 0.04. CR value less than 0.10 showed the consistency of the pairwise comparisons created for the “hardness and minerals” substances.

**Table 2:** The pairwise comparison matrix and parameter weights

Parameters	Acidity	Alkalinity	Ca hardness	Total hardness	Cl^−^	SO_4_^2−^	Weights
Acidity	1	1	3	1/3	1/9	3	0.08
Alkalinity	1	1	3	1/3	1/9	5	0.09
Ca hardness	1/3	1/3	1	1/5	1/9	1	0.04
Total hardness	3	3	5	1	1/3	7	0.21
Cl^−^	9	9	9	3	1	9	0.54
SO_4_^2−^	1/3	1/5	1	1/7	1/9	1	0.03

**Fig. 7:**
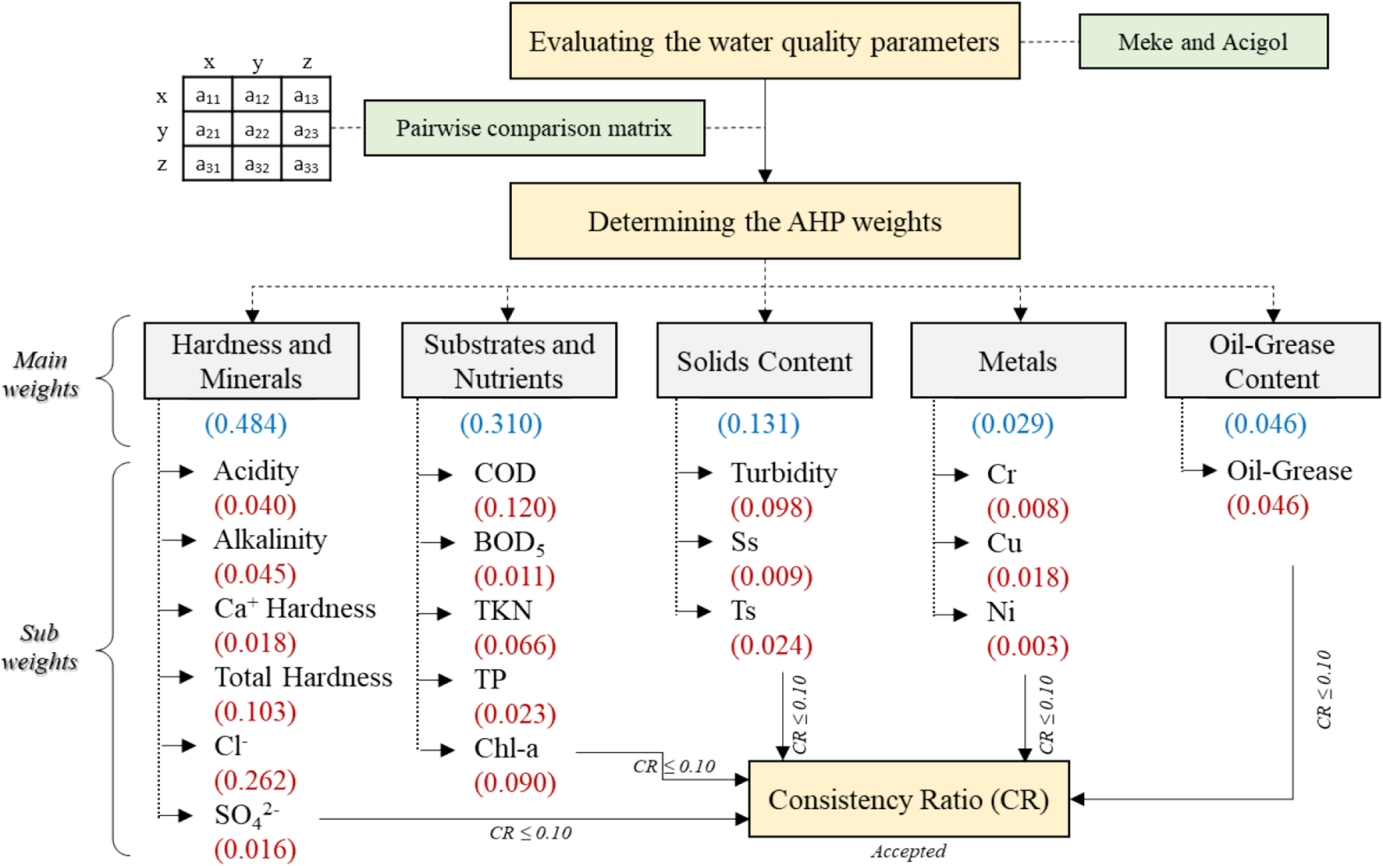
Main and sub-parameter weights in water quality

The weighting process was carried out similarly for 5 main parameters and 18 sub-parameters (Fig. 7). The coefficients in blue in Fig. 7 represent the weight of the main parameters, and the coefficients in red represent the weight of the sub-parameters. Since CR ≤ 0.10, it was concluded that the weights and comparison matrices of the parameters were consistent.

The most effective parameter group on lake water quality was “hardness and minerals” (0.484), and the second most important parameter group was “substrates and nutrients” (0.310). In terms of sub-parameter weights, Cl^−^ (0.262) in the “hardness and minerals” group and COD (0.120) in the “substrates and nutrients” group were found to be the highest. The lowest weights were seen in the metals group.

### Creating the group maps with indexing

**Fig. 8:**
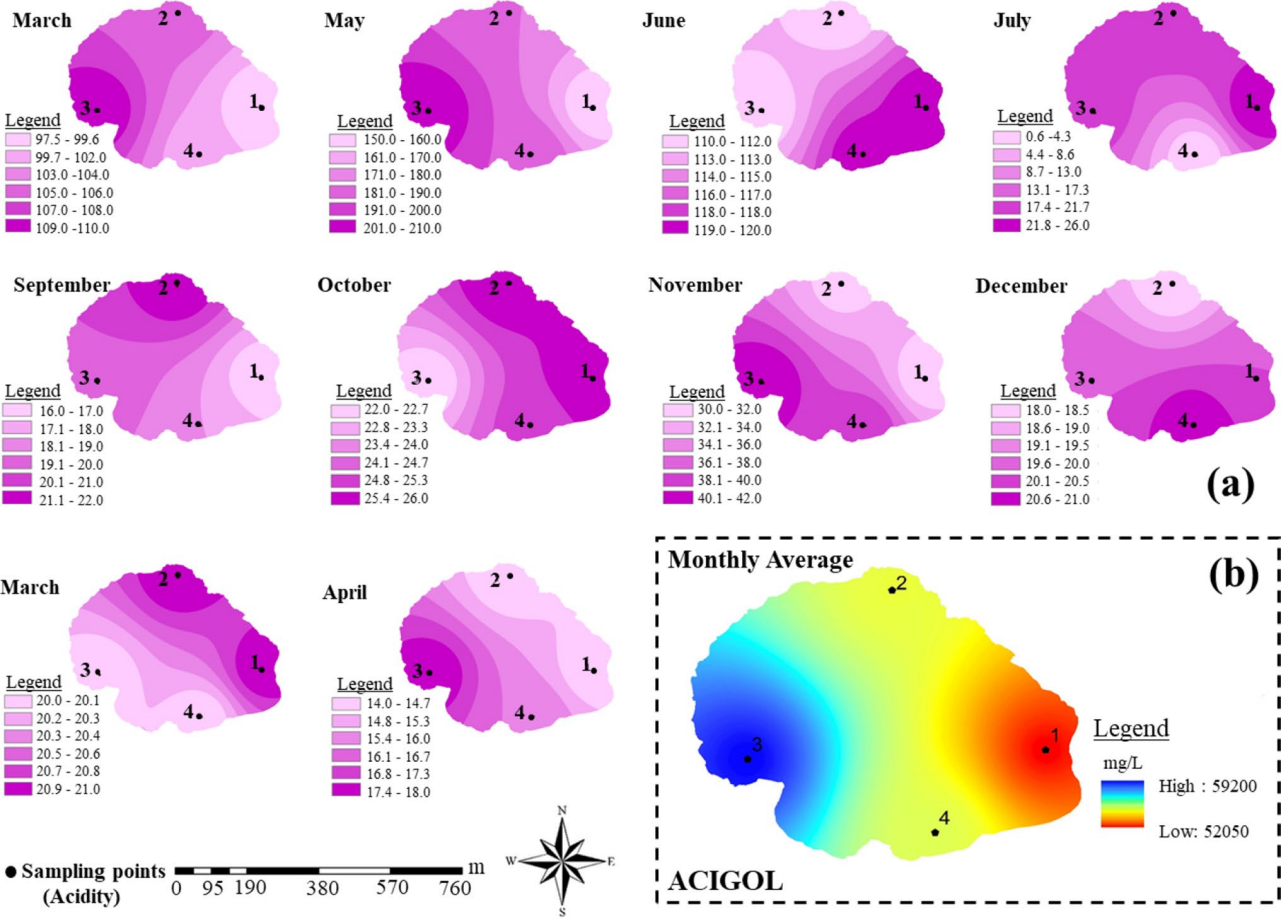
Integrated map showing (**a**) the time-location dependent variation of acidity in Acigol Lake and (**b**) the monthly average of acidity

A total of 360 spatial maps (18_parameter_ × 10_period_ × 2_lakes_) were produced using IDW for 18 water quality parameters measured in Acigol and Meke Lakes. However, not to increase the volume article, the 10 spatial and one integrated maps of the acidity parameter in Acigol Lake were presented as example of created maps (Fig. 8). Using spatial maps, acidity variation based on time and location was determined in Acigol Lake (Fig. 8a). In addition, the spatial maps were combined and the integrated map showing the monthly average of the parameter was interpreted (Fig. 8b). The spatial change of acidity values can also be clearly seen on the integrated map.

It is obvious that the lower the number of sample points, the higher the chance of inaccuracy and inconsistency in the dataset when deriving using interpolation or extrapolation or other methods. For accuracy assessment the maps were validated by comparing the measured parameter values from the literature with predicted intervals in this study. Table 3 indicates maximum-minimum value intervals, means, and standard deviations of experimental data of this study. Some parameter values of samples taken from points with known coordinates were collected from studies reported in the literature (including both papers and dissertations). The predicted values of this study corresponding to the reported coordinates in the literature were listed for all 10 sampling periods and their maximum-minimum value intervals, means, and standard deviations were calculated. The measured values of literature in different years and months were seen to fall this interval in almost all cases (Table 3). This comparison verified that the predicted values were consistent and valid. Therefore, the created maps were used for further steps of the proposed methodology.

**Table 3:** Verification of the modeled parameter values by comparing the predicted intervals with the measured parameter values in the literature

	Statistics of measured data in this study				Statistics of predicted data of this study at the sampling coordinates of the reference				The measured value reported in the cited reference	
	Max value	Min value	Mean	Std deviation	Max value	Min value	Mean	Std deviation		
Meke Lake										
Total Hardness	110,000	7000	51,930	25,474	95,544	22,878	53,207	22,901	48,800	Akdemir (2008)
Alkalinity	210,000	10,000	78,880	59,586	206,342	10,377	83,296	66,096	60,000	Yildiz and Balik (2005)
Ca	110,000	7000	51,930	25,474	45,071	8195	21,569	14,121	1691	Aycan (2012)
					45,071	8195	21,569	14,121	5690	Aycan (2012)
Cu	0.693	0.000	0.131	0.137	0.254	0.009	0.122	0.092	0.067	Asikkutlu et al. (2021)
Ni	0.590	0.000	0.131	0.160	0.419	0.003	0.139	0.130	0.056	Asikkutlu et al. (2021)
					0.419	0.003	0.139	0.130	0.32	Asikkutlu et al. (2021)
SO_4_	297,581	374	55,782	66,497	205,724	8452	53,955	56,058	20,000	Anonym (2006)
Cl	827,500	3500	149,540	183,254	614,301	28,391	150,967	161,456	188,000	Anonym (2006)
Acıgol Lake										
Total Hardness	98,500	21,000	43,313	22,744	96,339	21,518	43,012	21,989	27,700	Akdemir (2008)
Alkalinity	230,000	13,000	75,400	54,134	169,019	14,082	72,156	48,736	60,000	Yildiz and Balik (2005)
Ca	55,000	8000	19,338	12,482	35,097	11,217	18,415	8501	31,900	Aycan (2012)
Ni	0.456	0.000	0.215	0.103	0.450	0.083	0.240	0.122	0.170	Asikkutlu et al. (2021)

**Fig. 9:**
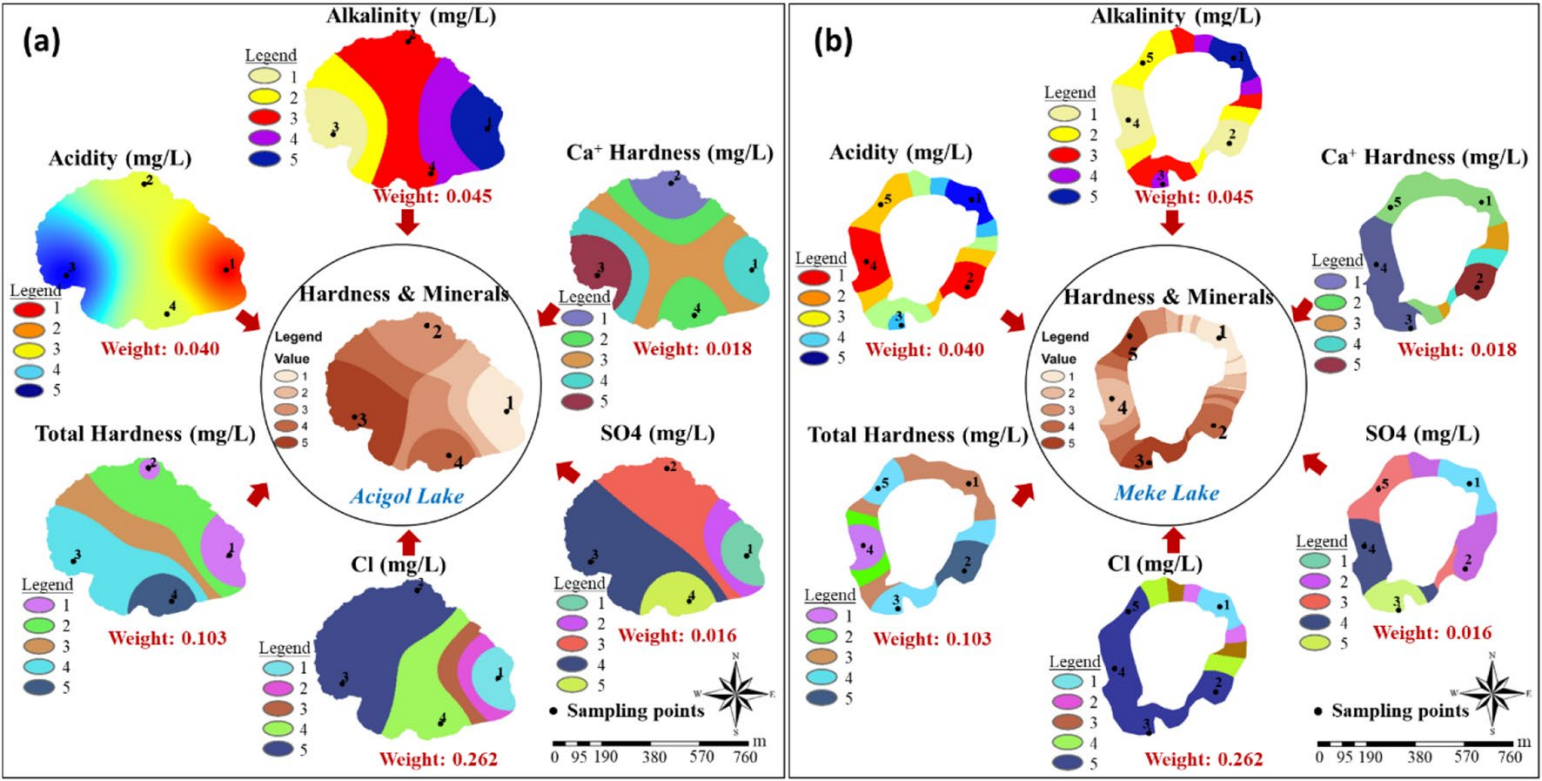
“Hardness and minerals” group maps: **a** Acigol Lake and **b** Meke Lake

**Fig. 10:**
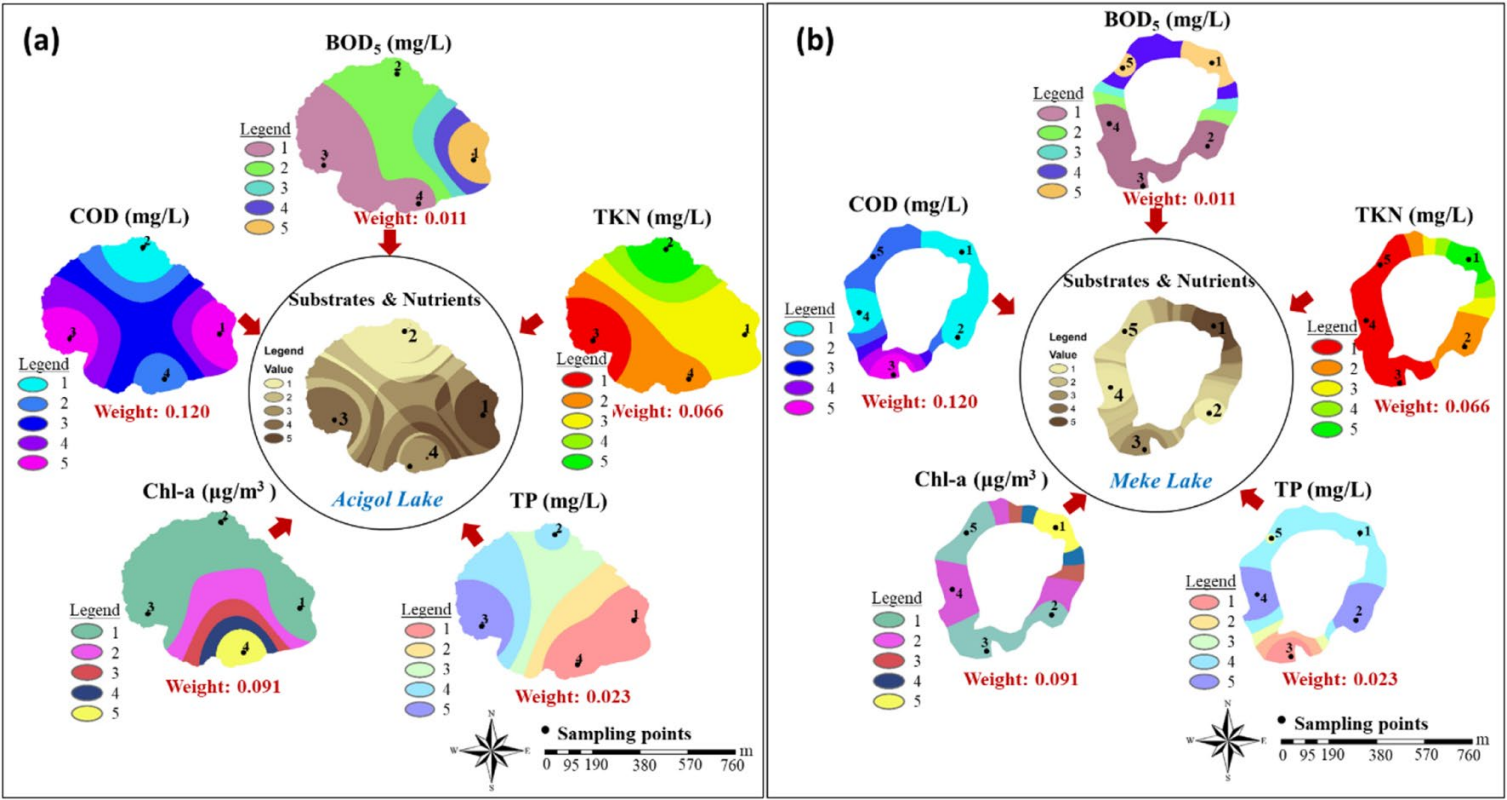
“Substrates and nutrients” group maps: **a** Acigol Lake and **b** Meke Lake

**Fig. 11:**
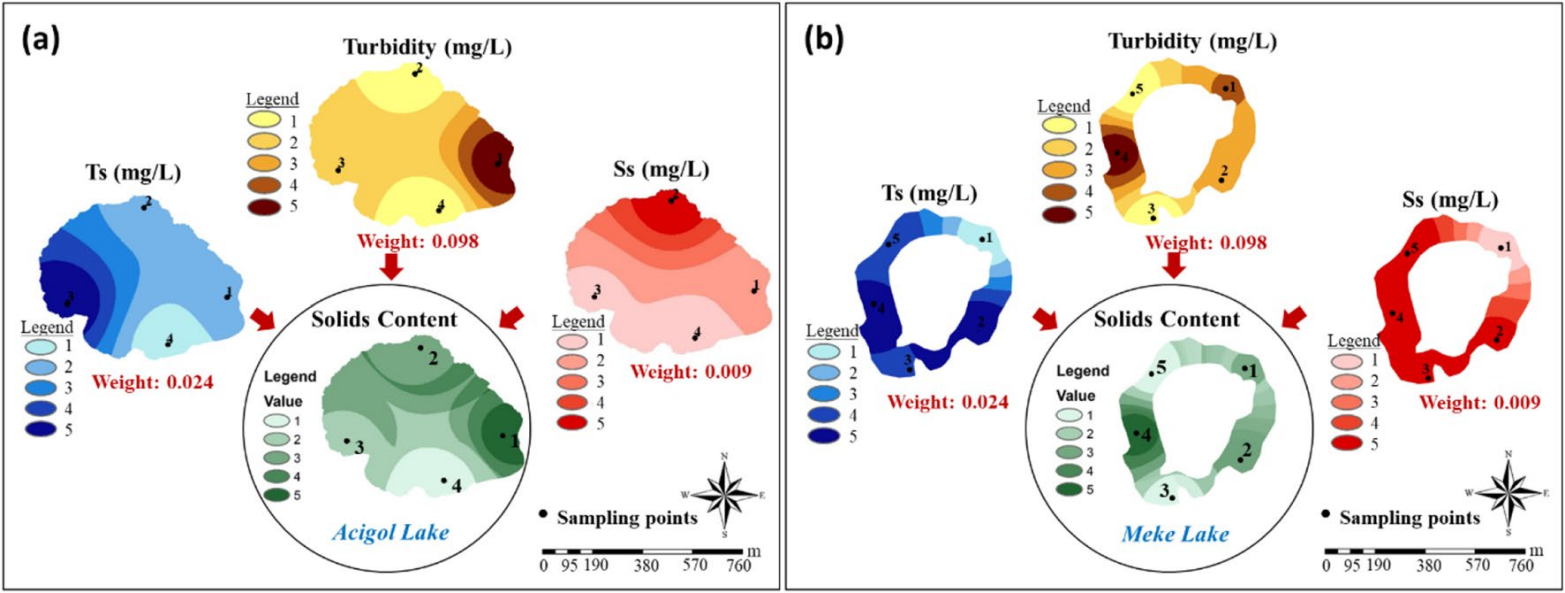
“Solids content” group maps: **a** Acigol Lake and **b** Meke Lake

**Fig. 12:**
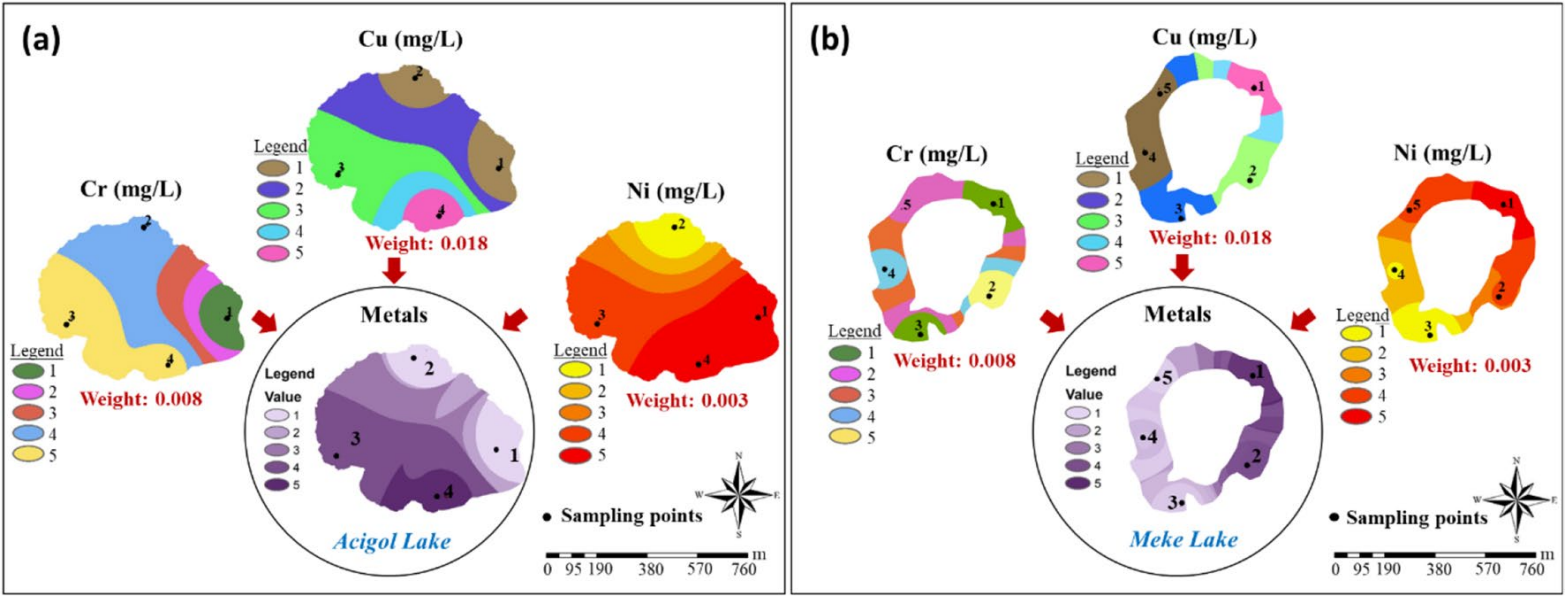
Metal group maps: **a** Acigol Lake and **b** Meke Lake

**Fig. 13:**
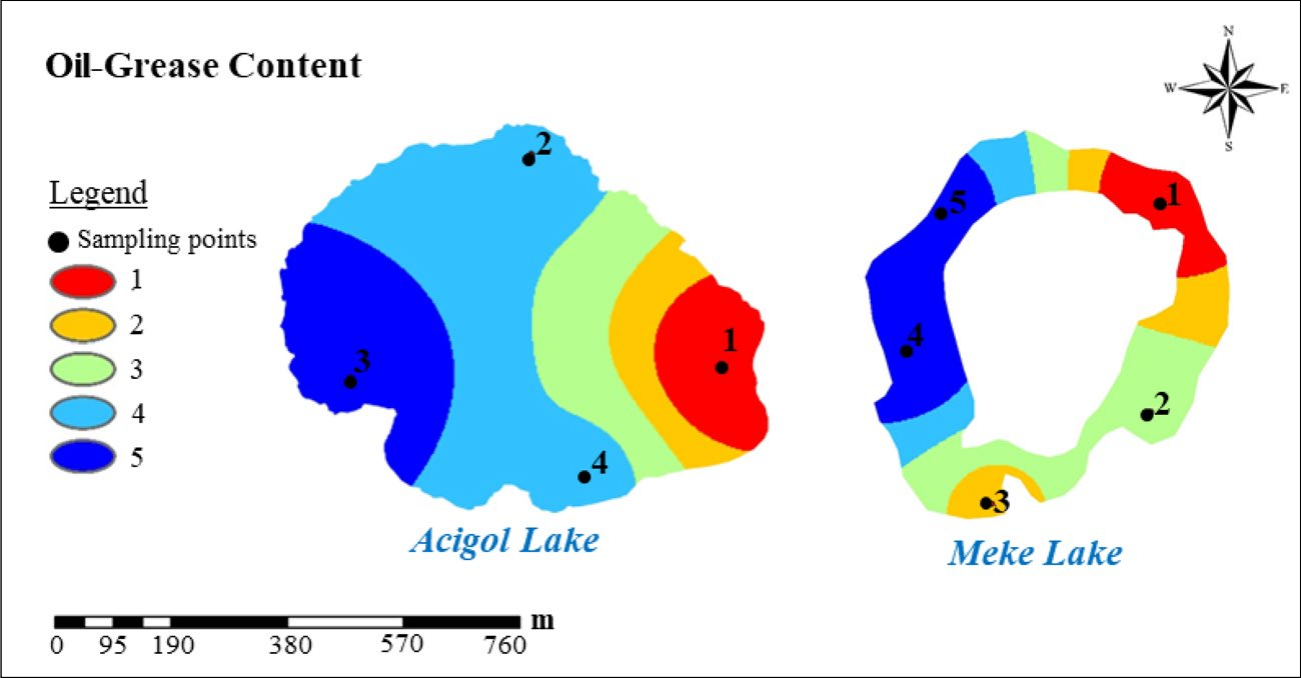
Oil-Grease content group maps

The integrated maps produced for all parameters were reclassified and divided into five classes, and group (index) maps were created by multiplying with AHP weights calculated in Fig. 7 (Figs. 9, 10, 11, 12, and 13). “Hardness and minerals” is the group with the more number of parameters compared to the others. This also affects the weight distribution of the parameters within the group. A meaningful and single result is reached when these multiple parameters were evaluated together and with their effect weights.

According to the results, “hardness and minerals” group parameters had the highest concentrations around point 3 in Acigol Lake (Fig. 9a), in regions 3 and 5 in Meke Lake (Fig. 9b), while they had the lowest concentration around point 1 in both lakes. Where acidity and alkalinity were the highest, total hardness, Cl^−^, and SO_4_^2−^ were the lowest, or vice versa. Actually, total hardness did not have the highest weight as seen in Fig. 7. However, as can be seen in Fig. 9, the total hardness value was as decisive as the ion concentration alone since different hardness compounds were in close concentrations in water quality. While the regions with the highest and lowest concentrations of acidity and alkalinity in Acigol Lake were opposite to each other, they were the same in Meke Lake. Since dolomitic structures (travertine) are dominant in the region, Mg is present, and therefore, the factor determining the water hardness is the total hardness parameter, which includes both Ca and Mg hardness. Since the pH was between 7 and 8, it was understood that there is no OH^−^ ion in the alkalinity content. Thus, the alkalinity originates from the bicarbonate and carbonate ions. The fact that the regions with the highest and lowest alkalinity and Cl^−^ and SO_4_^2−^ values were opposite each other confirms that the hardness of the lake waters was non-carbonate hardness.

In water quality, substrates are carbon source (either organic matter or inorganic C for autotrophs), and nutrients include nitrogen and phosphorus which are the main major components of a living cell. Although a wide variety of minerals and trace elements can be classified as nutrients, those required in most abundance by aquatic species are nitrogen and phosphorus. The substrate carbon is readily available from many sources as atmospheric CO_2_, alkalinity, organic matter decay products, etc. Nitrogen and phosphorus are the nutrients that are limiting factors in aquatic species growth. As bacterial species are not surviving in these lakes, only algal population had been checked and Chl*-a* parameter was followed. The COD parameter in the “substrates and nutrients” group had higher concentration in the east and west of Acigol, the two opposite regions, and in region 3 region in the Meke. While the “substrates and nutrients” parameters in both lakes were highest in region 1, they had the lowest concentrations in region 2 in Acigol Lake (Fig. 10a), and in regions 2 and 4 (Fig. 10b) in Meke Lake. The presence of COD and BOD_5_ parameters in the lakes indicates a human-induced organic pollution effect other than natural organics. The presence of nitrogen and phosphorus also supports this. Because the most important sources of these are animal wastes, wastewater, and fertilizers and pesticides used in agricultural areas. The region where algae were most abundant in Meke Lake was about the same as the region where nitrogen, which is the nutrient that algae consumes the most, was also high. On the other hand, in Acigol Lake, both COD and BOD_5_ are high in region 1, while COD is the highest and BOD_5_ is the lowest in region 3. COD was low and TKN was highest in region 2. Chl*-a* was high only in region 4 where not all substrate and nutrient parameters were high, while it was lower in other regions. The findings of this parameter, which represents the presence of algae population, in Acigol Lake differ from those in Meke Lake. However, TKN, TP, and Chl*-a* concentrations in Meke Lake were much higher than in Acigol Lake as can be remembered from Fig. 4, and since Acigol was a lake with a higher water depth, the light penetration required for algal growth would be much lower here. As a result, there was not any “substrates and nutrients” parameter with a clear dominance in group map formation due to the lack of a systematic change. However, anthropogenic effects can be said on lake-water quality.

“Solids content” was created as a parameter group that aims to evaluate the presence of both organic and inorganic particles and colloids in the lake water. These solids may originate naturally from rocks and microorganisms in the lake waters, or they may be transferred to the lake water from outside by anthropogenic effects. The most important effects are that they prevent the light penetration into the lake water and the access of microorganisms to the dissolved structures and even contribute to the formation of precipitates. The highest values of turbidity, Ss, and Ts parameters were observed at different points in Acigol Lake (sampling points 1, 2, and 3, respectively). However, apart from these points, they were not in very high concentrations throughout the lake (Fig. 11a). The highest values of turbidity, Ss, and Ts parameters in Meke Lake were in region 4, but Ss and Ts concentrations were also high in regions 2, 3, and 5 around this point. In the water of Meke Lake, the lowest Ss and Ts contents were observed in region 1, and the lowest turbidity was observed in regions 3 and 5 (Fig. 11b). In Acigol Lake, point 4 is the region with the lowest parameter values. In Meke Lake, the points where the lowest values of all parameters were observed were not the same and the lowest solid content was found at the same points with the turbidity in the group map. In the AHP results, the solid content was highest in region 1 in Acigol and in region 4 in Meke, since the parameter weight of turbidity was high. The regions with the highest Ts parameter, including dissolved solids, were similar to the regions with high Cl^−^ concentrations in both lakes, the most important basis of which was considered to be salinity.

In a natural water body, there are no heavy metals originating from nature unless there is a significant density of geological formations and rocks and solvent structures in the water content. In this case, it should be considered that any metal detected in the water is of anthropogenic origin (transported by water or air movement). In this study, Cr, Cu, and Ni were detected in water samples, mostly below 0.3 mg/L (Fig. 4). In Acigol Lake, the highest concentrations of Cr, Cu, and Ni were found in regions 1–4, and the lowest concentrations were found in region 1 for Cr and Cu and region 2 for Ni (Fig. 12a). In Meke, Cr, Cu, and Ni were densely located in region 1 (Fig. 12b). In the group maps prepared for the two lakes, it is noteworthy that the metal concentration is more prominent in a certain region of the lake and this is in the south-west direction in Acigol Lake and in the north-east direction in Meke Lake. As can be seen from the group map, Ni concentration especially increases in the direction of the wind on almost the same side of both lakes since metals are also dependent on atmospheric factors.

Oil-grease has poor solubility in water and has tendency to separate from the aqueous phase due to density diference. Oil-grease include hydrocarbons, esters, oils, fats, high-molecular-weight fatty acids, etc. These components can be released by organic matter and cell degradation, but the concentration generated in this way will be lower compared to external anthropogenic sources. Industrial activities, treatment sludge, agricultural machinery, and other machinery-using activities in the vicinity may have the potential to mix oil into the lake water. Since there is only one parameter in the oil-grease content group, the integrated and group map for both lakes was the parameter itself. Oil-grease content was found at the highest concentration in region 3 in Acigol Lake and in Meke Lake at points 4 and 5. The lowest oil-grease concentration was found at point 1 in both lakes (Fig. 13).

The locations with the highest and lowest raster values in the integrated maps (for each parameter) and the group maps presented in Fig. 9, 10, 11, 12, and 13 were determined and compared in Table 4. In terms of parameter evaluation, it was observed that the points 2 and 3 in Meke Lake and 3 in Acigol Lake for “hardness and minerals” group, point 1 in Meke Lake and point 1 in Acigol Lake for “substrates and nutrients” group, points 2 and 4 in Meke Lake for solids group, and point 4 in Acigol Lake for Metals group had more dominant concentrations. In terms of group maps, the highest raster values are in regions 3 and 5 for “hardness and minerals” group, and in region 1 for substrate and nutrients, metals in Meke Lake. In Acigol Lake, the highest raster values are in region 3 for “hardness and minerals” group, in region 1 for “substrates and nutrients” and solids content, and in region 4 for metals group (Table 4). From these findings, it was revealed that grouping and weighting the parameters by considering their effects on water quality is a very accurate approach.

**Table 4:** The lowest (L)/highest (H) raster values in the integrated and group maps

Parameters	Meke					Acigol			
	Sampling points					Sampling points			
	1	2	3	4	5	1	2	3	4
Acidity	H	L	H	L		L		H	
Alkalinity	H	L	H	L		H		L	
Ca hardness		H	L	L			L	H	
Total hardness	L	H		L		L	L	H	H
Cl^−^	L	H	H	H	H	L	H	H	
SO_4_^2−^	L		H	H		L		H	H
“Hardness and minerals” group map	**L**		**H**		**H**	**L**		**H**	
COD	L	L	H	L		H	L	H	
BOD_5_	H	L	L	L	H	H		L	L
TKN	H		L	L	L		H	L	L
TP		H	L	H		L		H	L
Chl*-a*	H	L	L		L	L	L	L	H
“Substrates and nutrients” group map	**H**	**L**				**H**	**L**		
Turbidity	H		L	H	L	H	L		L
Ss	L	H	H	H	H		H	L	L
Ts	L	H		H				H	L
“Solid content” group map			**L**	**H**	**L**	**H**			**L**
Cr	L	H	L			L		H	H
Cu	H			L	L	L	L		H
Ni			L	L	H	H	L		H
Metal group map	**H**		**L**		**L**	**L**	**L**		**H**
Oil and grease	L			H	H	L		H	
Oil-grease content group map	**L**			**H**	**H**	**L**		**H**	
Total #of H in parameters	7	7	6	7	5	5	3	10	6
Total #of L in parameters	8	5	8	9	4	9	7	5	6

### Final water quality maps of Acigol and Meke Lakes

**Fig. 14:**
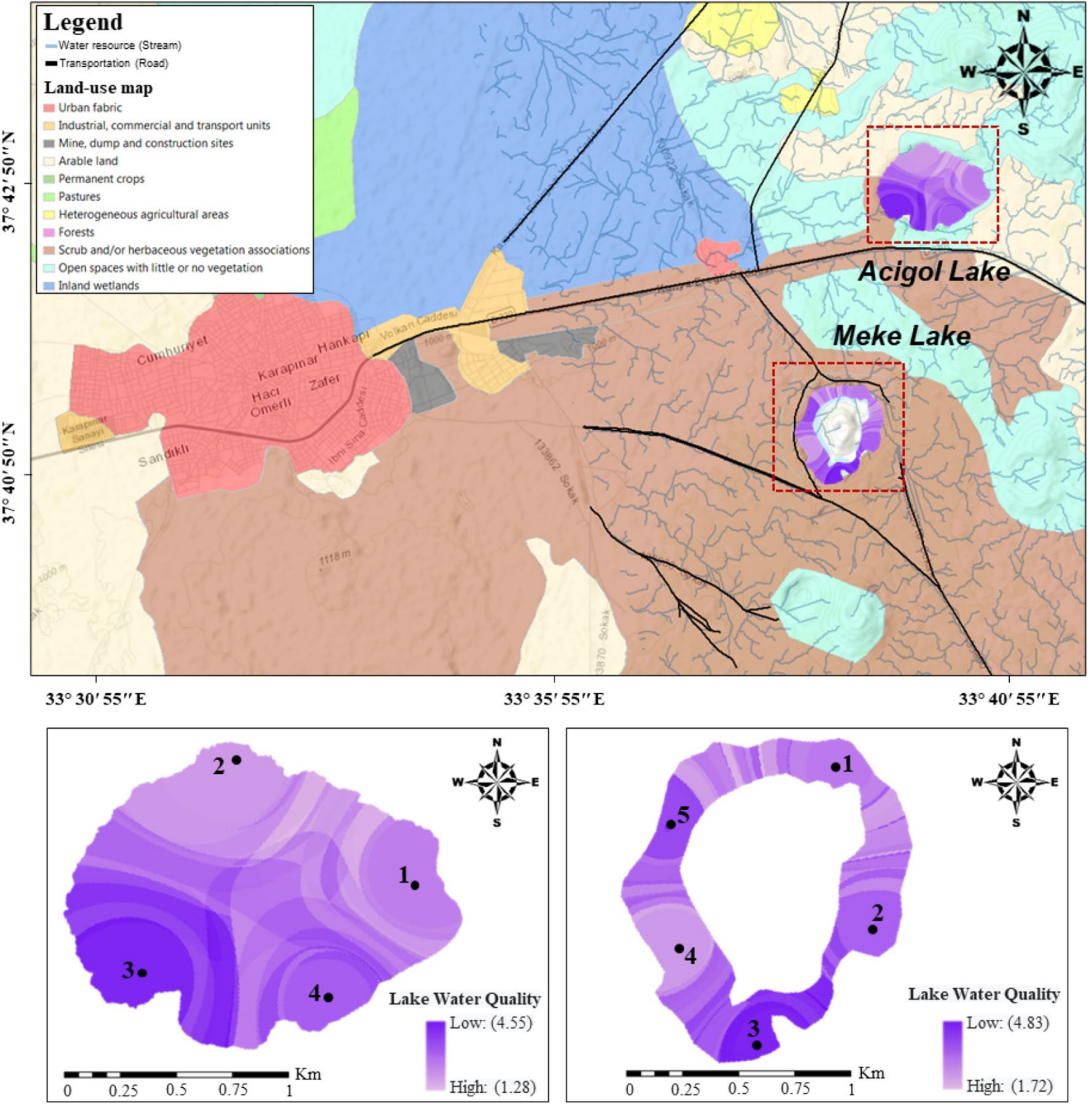
Association of lake water quality with land use

**Fig. 15:**
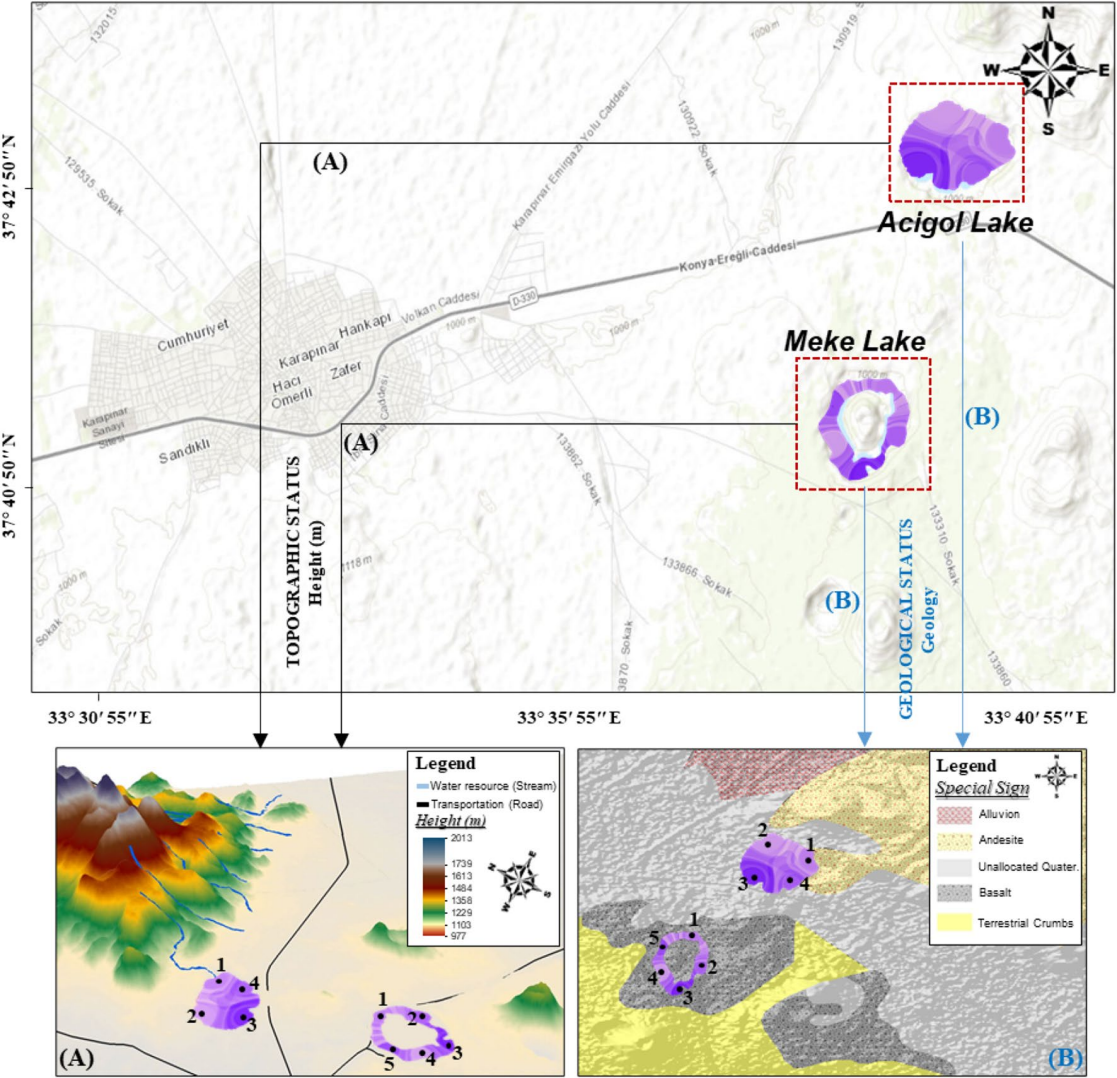
Association of lake water quality with topography and geology

In the final stage of the proposed methodology, lake water–quality maps obtained by combining the five group maps according to their AHP weights were related to other features of the region (Figs. 14 and 15). It was seen that the parameter values were higher in the south-west direction of Acigol Lake and in the south-southeast direction of Meke Lake. In the previous stage, it was interpreted that this may be partly due to geological structure and partly due to anthropogenic origin. There is sparse vegetation and a water source in the east of Acigol. The water quality of this area has lower pollution compared to other areas close to non-irrigated arable land and forests. The lake-water quality was low in the area of Acigol close to the motorway, and even had quite high parameter values (Fig. 14). The facility, which is located on the side of the road including a gas station, has an effect on this. In addition, there is a residential area in the southwest of the lake and agricultural activities in the north.

The topography of Acigol is higher in the south, with a decreasing slope toward east–west-north. Meke Lake and its surroundings are basalt, and the bottom of the Acigol region is andesite (Fig. 15). Volcanic rocks consisting of lava flows, ashes, tuffs, cinder cones, maar pyroclastics, and olivine basalts are distributed in the south, east, and northeast of Karapınar. Sultaniye Plain is a old quaternary, the depth of the graben is 250–300 m, and the Quaternary stack filling the graben consists entirely of gray plastic clays. White colored Neogene marl and limestones are on the ground (Özgüner 1993). Water rich in Ca(HCO_3_)_2_ formed travertine cones by precipitating CaCO_3_ on the earth. Travertines observed around Acigol are dark yellow, beige color and porous (Nazik et al. 2004). Travertines are calcium carbonate deposits that precipitated as a result of the decrease in CO_2_ pressure of waters rich in Ca^+2^ and CO_3_^2−^. Geological, tectonic, hydrological, geomorphological, climatological, and biological conditions have different effects on their formation. It is thought that their contribution to “hardness and minerals” and “solid content” in the Acigol Lake water is very high.

Hypersaline environments can be found on all continents and in many countries. These environments fall into two basic categories. The salt composition of the lakes, called thalassohalin, is similar to the salt composition of sea water, and the sodium and chloride concentrations are similar to sea water (Aycan 2012). As a result of the evaporation of sea water, these environments can be alkaline or neutral. As evaporation continues, minerals such as gypsum precipitate. When NaCl reaches the saturation point, bright red crystallized ponds may form (Oren 2002). The compositions of hypersaline ponds are significantly affected by human activities and their chemical-biological structure can change. For this reason, when the concentration of any pollutant parameter is found in salt water, it can create much higher pollution than its effect in freshwater. A thalassohaline hypersaline environments have a completely different salt composition than seawater. In such an environment, the concentration of divalent cations (1.9 M Mg^+2^, 0.4 M Ca^+2^) is higher than the concentration of univalent (1.6 M Na^+^, 0.14 M K^+^) cations. In addition, the pH is relatively lower (Oren 2002). The effects of “substrates and nutrients” and metal parameters, which represent anthropogenic pollution in both lake waters, are associated with this situation, because the closest settlement around the lakes is 8 km away. Although the settlement impact is not direct, there are still humanitarian activities around.

As a result of drought, reduced precipitation and inflows can result in increased concentrations of parameters. Changes in precipitation may affect the flow magnitude, thereby changing the nature and quantity of inputs from surrounding area to lakes through the leaching and erosion of soils, changes in microbial transformation rates of particulate, and dissolved organics and through changes in catchment vegetation. Meke Lake is very shallow and has a high salt content that can reach up to 32% in dry seasons (Aycan 2012). In 2010, 10.4 g/L Na^+^, 3.9 g/L K^+^, 0.3 g/L Ca^+2^, 120 g/L Mg^+2^, 200 g/L SO_4_^2−^, and 188 g/L Cl^−^ were detected in the lake water (Karaaslan 2010). The feeding of Acigol Lake is provided from underground. The water tastes bitter because it is salty and carbonated (Akkoz et al. 2009; Aycan 2012). In 2011, salinity was reported as 6–6.5% (pH: 7.6–8.4) in Acigol water and 18–25% (pH: 7–8) in Meke water. It was observed that the pH increased slightly when the salinity decreased (Aycan 2012). After these studies, no further studies on lake water quality have been carried out since the water in these two lakes decreased with drought.

In a study, Wang et al. (2021) reported as Ss = 5–100 mg/L; DO = 2–9 mg/L; BOD_5_ = 0–12 mg/L; COD = 30–150 mg/L; TP = 0.1–1.0 mg/L; TN = 2–6 mg/L for a hyper-eutrophic Lake in China. The worst lake water quality in Chinese standards was defined as DO < 1 mg/L, COD = 61 mg/L, NH_3_–N = 4.25 mg/L, TKN = 14 mg/L, and TP = 0.82 mg/L (Yao et al. 2021). DO, COD, NH_3_-N, TKN, and TP average concentrations of lake water were 7.77, 18.11, 0.31, 1.06, and 0.05 mg/L, respectively. Among the various lakes in China, the maximum lake water Chl*-a*, COD, TKN, and TP concentrations were detected as 0.262, 13.6, 16.05, and 0.5 mg/L (Yao et al. 2021). All the parameter concentrations observed in this study were worse than all these values. The samples were of much lower DO yet much higher COD, BOD_5_, Ss, and nutrient concentrations. Growth in nitrogen and calcium concentrations suggests intensified activities utilizing products rich in nutrients while the decrease in average DO concentration to less than 1 mg/L implies increased discharge of oxygen-demanding wastes in the KCB. In order to evaluate the nutrient levels in lake water, the Redfield ratio (TN/TP = 7.2 (Redfield 1958)) ratio can be used, and in this study, it was calculated as 28.2 for Meke and 12.3 for Acigol Lakes, both are well above 7.2 (Wang et al. 2021). This implies limitation of both N and P; the lake waters were P limited for microbial growth rather than N + P limited (Qin et al. 2020). The decrease in TN/TP ratio can be correlated with increased Chl*-a* (Liang et al. 2020).

## Conclusion

The following conclusions and recommendations can be presented by this study:

- This study indicated that, for shallow lakes, four to five homogenously distributed sampling points for 0.5–1.2 km^2^ surface area can be used to create the predicted whole lake water quality maps for the estimation of rough water quality. GIS mapping is a tool that can be used successively for this purpose.
- The parameter changes in different locations of the lake for a year period can be analyzed through IDW. According to the time-dependent spatial results, produce spatial maps for each parameter, categorize parameter maps (hardness and minerals, substrates and nutrients, solids content, metals, and oil-grease), create group maps, assign parameter effect weights in groups for revealing multiple relationships, and finally, evaluate lake water quality maps by combining group maps according to AHP weights. This can be a suggested methodology to roughly evaluate lake-water quality from limited number of data.
- The obtained final map is the integrated form of predicted data and it summarizes the situation inside the lake. The effect of external pollution sources, surrounding land use, geological structure, environmental factors, and anthropogenic factors in the region on the water quality of the lakes can be investigated by evaluating these factors together with final map. This proposed approach was investigated with the data of Acigol and Meke Lakes in Turkey.
- The two lakes in this study have ecological, hydromorphological, and socio-economical importance in the KCB. The maps of Acigol and Meke obtained as a result of the study can be used in spatial decision support of administrative management. The effect of new establishment, settlement, or any land use change on the water quality of lakes can also be monitored on the final maps. Moreover, the results of this study are supportive for Meke and Acigol Lakes in Karapınar/Konya district to be geoparks.
- The proposed approach is flexible and applicable to any lake-water-quality assessment data. Increasing the number of sampling points and sampling periods will certainly increase the performance of the parameter prediction and final map accuracy by decreasing the standard deviations. However, this study indicated that, even with a limited number of data, the whole lake-water-quality maps can be created.
- This methodology will contribute to the environmental management efforts on site where detailed analytical data collection is not feasible or convenient.

## Data Availability

The datasets generated during and/or analyzed during the current study are available from the corresponding author on reasonable request.
